# p,p’-DDT induces testicular oxidative stress-induced apoptosis in adult rats

**DOI:** 10.1186/s12958-017-0259-0

**Published:** 2017-05-26

**Authors:** Neila Marouani, Dorsaf Hallegue, Mohsen Sakly, Moncef Benkhalifa, Khémais Ben Rhouma, Olfa Tebourbi

**Affiliations:** 1 Laboratory of Integrated Physiology, Faculty of Sciences, Carthage University Tunisia, Bizerte, Jarzouna Tunisia; 2 Reproductive Medicine and Medical Cytogenetics Department, Regional University Hospital and School of Medicine, Picardie University Jules Verne, Amiens, France

**Keywords:** p,p’-DDT, Testis, Oxidative stress, Apoptosis, DNA fragmentation, Rat

## Abstract

**Background:**

The 1,1,1-trichloro-2,2-bis(4-chlorophenyl)ethane (p,p’-DDT) is a known persistent organic pollutant and male reproductive toxicant. The present study is designed to test the hypothesis that oxidative stress mediates p,p’-DDT-induced apoptosis in testis.

**Methods:**

Male Wistar rats received an intraperitoneal (ip) injection of the pesticide at doses of 50 and 100mg/kg for 10 consecutive days. The oxidative stress was evaluated by biomarkers such lipid peroxidation (LPO) and metallothioneins (MTs) levels. Antioxidant enzymes activities was assessed by determination of superoxide dismutase (SOD), catalase (CAT) and hydrogen peroxide (H_2_O_2_) production. In addition, glutathione-dependent enzymes and reducing power in testis was evaluated by glutathione peroxidase (Gpx), glutathione reductase (GR), glutathione S-transferase (GST) activities and reduced and oxidized glutathione (GSH - GSSG) levels. Apoptosis was evaluated by DNA fragmentation detected by agarose gel electrophoresis. Germinal cells apoptosis and the apoptotic index was assessed through the TUNEL assay.

**Results:**

After 10 days of treatment, an increase in LPO level and H_2_O_2_ production occurred, while MTs level, SOD and CAT activities were decreased. Also, the Gpx, GR, GST, and GSH activities were decreased, whereas GSSG activity was increased. Testicular tissues of treated rats showed pronounced degradation of the DNA into oligonucleotides as seen in the typical electrophoretic DNA ladder pattern. Intense apoptosis was observed in germinal cells of DDT-exposed rats. In addition, the apoptotic index was significantly increased in testis of DDT-treated rats.

**Conclusions:**

These results clearly suggest that DDT sub-acute treatment causes oxidative stress in rat testis leading to apoptosis.

## Background

The 1,1,1-trichloro-2,2-bis(4-chlorophenyl)ethane (p,p’-DDT) was commercialized as an agricultural pesticide in 1945. It is the first widely used synthetic organochlorine pesticide introduced all over the world to eliminate unwanted pests, and helped one billion people live free from malaria [1, 2]. It was banned for agricultural use in 1970s–1980s primarily on the basis of ecological consideration [1]. When DDT emissions ceased in 1990, about 634 kt DDT were released into the environment [3]. Even though the Stockholm Convention on Persistent Organic Pollutants listed DDT as the “Dirty Dozen” in 2001 for the global community [4], DDT is still currently used in indoor residue spraying in 14 tropical countries and several other countries are preparing to reintroduce it [5]. High levels of DDT (parts per million levels) were always detected in malaria control area. In South Africa, for example, the mean DDT concentration approached 7.3 mg/g in human serum and 240 mg/kg in chicken fat [6]. Also, numerous analytical studies showed higher levels of DDT and its main metabolite 1,1-dichloro-2,2-bis(4-chlorophenyl)ethane (p,p’-DDE) than the allowable daily intake in food [7], adipose tissues [8] and maternal milk [9] all over the world. The toxic effects of direct exposure of DDT in humans have been reviewed [10] and include endocrine disruptions [11], neurological diseases [12], cancer [13], reproductive diseases [14] and developmental abnormalities [15]. Studies have also shown that exposure to DDT provoke birth defects in wildlife [16]. A decreased testis weight, sperm cell count and motility as well as increased follicle stimulating hormone (FSH) and luteinizing hormone (LH) serum concentrations were observed in rats treated with DDT [17]. Also, a pronounced alteration of spermatogenic process with dramatic reduction of spermatozoa produced in the lumen of seminiferous tubule was observed in rats exposed to DDT [17]. On the other hand, it has been reported that oxidative stress can be used as a biomarker to evaluate damages and a possible mechanism of DDT and DDE toxicity in humans [18, 19]. Furthermore, oxidative stress is one of the best known causes of cellular damage, mostly due to the formation of free radicals that damage cell DNA [20]. In the testis, reactive oxygen species (ROS) generation might play a critical role in the initiation of p,p’-DDE-induced apoptosis in rat Sertoli cells through mitochondria-mediated pathway [21]. However, the mechanisms of the reproductive effects of DDT are also poorly understood. In the background of the existing information, it is hypothesized that exposure to p,p’-DDT would disrupt testis function in rat by inducing oxidative stress. Therefore, the aim of this work is to investigate the effect of p,p’-DDT subacute treatment on rat testis and the implication of oxidative stress and apoptosis in this organ. To this end, the status of the oxidative stress was evaluated by biomarkers such lipid peroxidation (LPO) and metallothioneins (MTs) levels. Antioxidant enzymes activities were measured such as superoxide dismutase (SOD), catalase (CAT) and hydrogen peroxide (H_2_O_2_) production. In addition, glutathione-dependent enzymes and reducing power in testes were evaluated. The characteristic DNA migration patterns of testicular tissues and the detection of apoptotic cells by TUNEL assay in germinal cells were aimed to be examined.

## Methods

### Animals and reagents

Male Wistar rats (50 days of age) were purchased from the Tunisian Company of Pharmaceutical Industries (SIPHAT, Rades, Tunis, Tunisia). The rats were housed under controlled conditions of temperature (25 °C) with a constant day/night cycle (light from 8:00 to 20:00). Food and water were provided ad libitum. DDT (98% pp’) were purchased from Sigma Chemical (St. Louis, MO, USA). Rats were randomized into three experimental groups of approximately similar weight (*n* = 8) as follows: (1) animals received daily an intraperitoneal (ip) injection of DDT diluted with corn oil at a dose of 50mg/kg body weight (b.wt) during 10 days, (2) animals were administered 10 daily injections of 100 mg DDT/kg b.wt, (3) control group received equal daily volumes of vehicle during the treatment period. The choice of the dosing period and DDT doses was based on the results of previous studies [17, 22, 23]. Rats were fed and observed daily. The body weight of rats was determined daily through the experiment. After 10 days of treatment, all animals were killed by decapitation, the left testis were dissected and weighed.

### Tissue preparation

Fractions of testicle (400 mg) from control and treated groups were homogenized in phosphate-buffered saline (PBS, pH 7.2). The homogenates were centrifuged at 600 x g for 10 min and recentrifuged at 13,000 x g for 20 min at +4 °C to obtain a postnuclear homogenate and postmitochondrial supernatant fraction [24].

### Malondialdehyde assay

Lipid peroxidation was measured in the testicle using thiobarbituric acid reacting substance (TBARS) following the method of Buege and Aust [25]. Briefly, the stock solution contained equal volumes of trichloroacetic acid 15% (w/v) in 0.25N hydrochloric acid and 2-thiobarbituric acid 0.37% (w/v) in 0.25N hydrochloric acid. One volume of the test sample and two volumes of stock reagent were mixed in a screw-capped centrifuge tube, vortexed and heated for 15 min on a boiling water bath. After cooling on ice the precipitate was removed by centrifugation at 1000 x g for 15 min and absorbance of the supernatant was measured at 532 nM against blank containing all the reagents except test sample. A standard curve was constructed extrapolating the amount of commercially bought product malondialdehyde (MDA) to the measured absorbance. The value is expressed in μmole of MDA formed per mg protein.

### Measurement of metallothioneins

Determination of MTs was performed according to the technique described by Eaton and Cherian [26]. Quantification of MTs was performed by using ^109^Cd. Briefly, a fraction (about 0.5g) of the tissues was homogenized in 1ml of a 0.25M sucrose solution. The homogenate were centrifuged at 10,000g for 10 min at 4°C, the supernatant was stored at−80 °C for analysis of MTs protein. 200 μl of ^109^Cd solution (2 μg/ml) were mixed with 200 μl of sample (heat-denatured supernatant) and allowed to incubate at room temperature for 10 min. Then 100μl of a 2% bovine haemoglobin solution were added to the tubes, mixed and heated in a 100°C boiling water bath for 2min. The tubes were placed on ice for several minutes, and centrifuged at 10,000g for 2 min in a microfuge and another 100μl aliquot of 2% haemoglobin was added. Heating, cooling and centrifugation were repeated once again. A 500μl aliquot of the supernatant fraction should be carefully removed. Lastly, aliquots of 500 μl of the supernatant were recovered carefully and transferred into clean tubes for counting their radioactivity.

### Determination of antioxidant enzymes activities

#### Superoxide dismutase activity

The method described by Murklund and Murklund [27] was used for assay of superoxide dismutase (SOD) activity. Briefly, the assay mixture contained 2.4 ml of 50 mM Tris–HCl buffer containing 1 mM EDTA (pH 7.6), 300 μl of 0.2 mM pyrogallol and 300 μl enzyme source. The increase in absorbance was measured immediately at 420 nm against blank containing all the components except the enzyme and pyrogallol at 10 s intervals for 3min on a Systronics Spectrophotometer. The enzyme activity was expressed as nmole pyrogallol oxidized per minute per mg of protein.

#### Catalase activity

The catalase activity was measured according to the method of Aebi [28]. Activity was assayed by determining the rate of degradation of H_2_O_2_ at 240nm in 10 mM of potassium phosphate buffer (pH 7.0). An extinction coefficient of 0.036mM/cm was used for calculations. The enzyme activity was expressed as μmol of H_2_O_2_ consumed per minute per mg of protein.

#### Hydrogen peroxide production

H_2_O_2_ generation was assayed by the method of Pick and Keisari [29]. Briefly, the incubation mixture contained 1.641 ml phosphate buffer (50 mM, pH 7.6), 54 μl horse radish peroxidase (8.5 units/ml), 30 μl of 0.28 nM phenol red, 165 μl of 5.5 nM dextrose, and 100 μl of enzyme source, incubated at 35°C for 30 min. The reaction was terminated by the addition of 60 μl of 10 N sodium hydroxide. The absorbance was read at 610 nM against a reagent blank on a Systronics Spectrophotometer. The quantity of H_2_O_2_ produced was expressed as nmol of H_2_O_2_ generated per mg of protein.

### Glutathione dependant enzymes and reducing power

#### Glutathione peroxidase activity

Gpx generation was assayed by the method of Paglia and Valentine [30]. The assay mixture contained 1.59 ml of phosphate buffer (100 mM, pH 7.6), 100μl of EDTA (10 mM), 100μl of sodium azide, 50 μl of glutathione reductase, 100μl of reduced glutathione 100μl of NADPH (200 mM), 10 μl of H_2_O_2_ and 10 μl enzyme source. Disappearance of NADPH was measured immediately at 340 nm against blank containing all the components except the enzyme at 10s intervals for 3min on a Systronics Spectrophotometer. One unit was defined as 1nmole of NADPH oxidized per minute and the specific activity was reported as units per mg of protein.

#### Glutathione reductase activity

GR activity was determined as described by Calberg and Mannervik [31]. In this assay, glutathione oxidized is reduced by GR at the expense of NADPH consumption, which is followed at 340 nm. GR activity is proportional to NADPH decay. GR activity was expressed as units per mg of protein.

#### Glutathione S-transferase activity

GST activity was measured using the method of Habig et al. [32]. Briefly, 1 mM of 1-chloro-2,4-dinitrobenzene (CDNB) was added to buffer containing 1 mM GSH and an aliquot of sample to be tested. Upon addition of CDNB, the change in absorbance at 340 nm was measured as a function of time. The extinction coefficient for this reaction is 9.6 mM^−1^cm^−1^. GST activity was expressed as μmol CDNB conjugates/min/mg protein and was reported as units per mg of protein.

#### Determination of reduced and oxidized glutathione

The levels of reduced and oxidized glutathione (GSH and GSSG) were estimated as described by Hissin and Hilf [33]. Briefly, GSH in the acid soluble supernatant fraction of testicular cells was reacted with *o-*phthaldialdehyde at pH 8.0 to yield a highly fluorescent cyclic product, while GSSG was determined by the same reagent but at pH 12 and in the presence of N-ethylmaleimide. GSH and GSSG contents were expressed as nmol per mg protein, which allowed the calculation of the glutathione redox ratio (GSSG/GSH).

### DNA-fragment extract and electrophoresis

DNA extraction and electrophoresis have been performed according to the method described by Ichimura et al. [34]. Briefly, Testis were rapidly frozen in liquid nitrogen and gently homogenized in cell lysis buffer (5mM Tris–HCI, 20mM EDTA, 0.5% Triton X100, pH 8.0) with a Polytron homogenizer. The homogenate was centrifuged at 2,500 rpm for 10 min. The supernate was suspended and centrifuged at 13,000 rpm for 10 min to remove high molecular DNA and cellular debris. RNase was added to the supernatant (10μg/ml) to react at 25° C for 30min, then Proteinase K (0.3 mg/ml) to react at 55° C for 60 min. This supernatant was mixed with an equal volume of phenol–chloroform–lsoamylalcohol, centrifuged at 9000 rpm for 10 min, and the uppermost layer was collected in a new vial. DNA fragments were collected from this layer, added with sodium acetate (0.3 M) and ethanol (70%), and centrifuged at 15,000 rpm for 30 min. DNA concentrations were determined by spectrophotometric absorption at 260nm. 4 μg of DNA/sample was loaded into 1.5% agarose gel in 89mM Tris, 89mM boric acid and 2.5 mM EDTA (pH 8.0), and was electrophoresed at constant current (90mA) for 3 h. The DNA bands were visualized using UV illumination (260 nm) after ethidium bromide staining, using a camera equipped with a Polaroid type 667 film with an orange filter (Kodak).

#### Detection of apoptotic cells by TUNEL assay

The testicular tissues were fixed overnight at room temperature by direct immersion in 4% paraformaldehyde in 0.1M phosphate buffer, pH 7.4. The samples were dehydrated with ethanol and toluene and embedded in paraffin wax. Serial sections (4 μm thick) were mounted on gelatin-coated glass slides and stained with TUNEL (TdT-mediated dUTP-digoxigenin Nick and Labeling). After deparaffinization and rehydratation, tissues sections were incubated with 0.1% (v/v) Triton X-100 for 2min on ice, followed by washing of the slides twices in PBS (CaCl_2_ 2H_2_O 0.8mM, KCl 2.6mM, KH_2_ PO_4_ 1.4mM, MgCl_2_ 6H_2_O 0.4mM, NaCl 136mM, Na_2_HPO_4_ 8 mM, pH 7.2). The specimens were then incubated one hour at 37°C in a solution consisting of 1mM cobalt chloride, 140 mM sodium cacodylate and terminal deoxyribonucleotidyl transferase (TdT) at a final concentration of 0.1U/μl to insert biotin-16-dUTP at the 3’ – ends of DNA fragments. A streptavidin-peroxydase complex and 3-amino-9- ethylcarbazole served as the detection system for biotin. The slides were washed in PBS, developed with 0.05% diaminobenzidine, and stained for 15 minutes at room temperature. Sections were lightly counter-stained with hematoxylin and mounted in glycerin jelly. Negative control included omission of TdT from the labeling mixture. The positively labeled cells appear as darkly brown stained. Apoptotic and normal cell numbers in 10 tubules in each slide were counted using Image-Pro Plus version 4.5 software (Media Cybernetics Inc, Silver Spring, MD, USA) at x 400 magnification. Apoptotic index was calculated as total TUNEL positive spermatogenetic cell number divided by total normal spermatogenetic cell number [35].

### Statistical analysis

Data were analyzed using Statistica for Windows version 5.0 Software. Overall differences in mean values between control and treatment groups were measured using one-way analysis of variance (ANOVA) followed by Turkey’s multiple comparison as the *post hoc* test. The results were expressed as means ± standard errors of the mean (SEM) and differences were considered statistically significant at *p* < 0.05.

## Results

### MDA and MTs levels

**Fig. 1:**
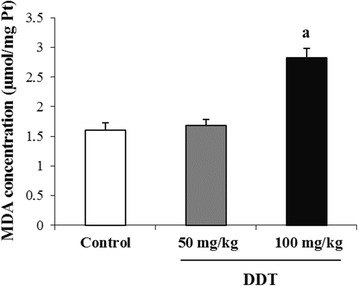
Effect of DDT on the concentration of malondialdehyde in testis of adult rat. Each value is the mean ± SEM of 8 determinations in duplicate per group. DDT treatment was preformed as described in the methods section. ^a^ : *p* < 0.01 compared with controls (Tukey’s multiple comparison post hoc *test)*

**Fig. 2:**
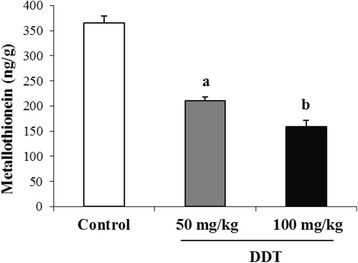
Effect of DDT treatment on testis metallothionein concentration in adult rat. Each value is the mean of 8 determinations with standard error (SEM). DDT treatment was preformed as described in Methods. ^a, b^ : *p* < 0.001 compared with controls (Tukey’s multiple comparison post hoc test)

Exposure of rats to DDT for 10 consecutive days induced a significant increase in MDA concentration in testis at the high dose (100 mg of DDT/kg). This increase was about 75.5% compared with the control group (Fig. 1). In contrast, the level of MTs in testis was significantly decreased in treated rats compared to control group (Fig. 2). The MTs levels were 209.99 ± 8.41 and 159.56 ± 12.56 ng/g, respectively, in rats receiving 50 and 100 mg of DDT/kg compared with 365.56 ± 13.54 ng/g in the control group.

### Antioxidant enzyme activities

**Fig. 3:**
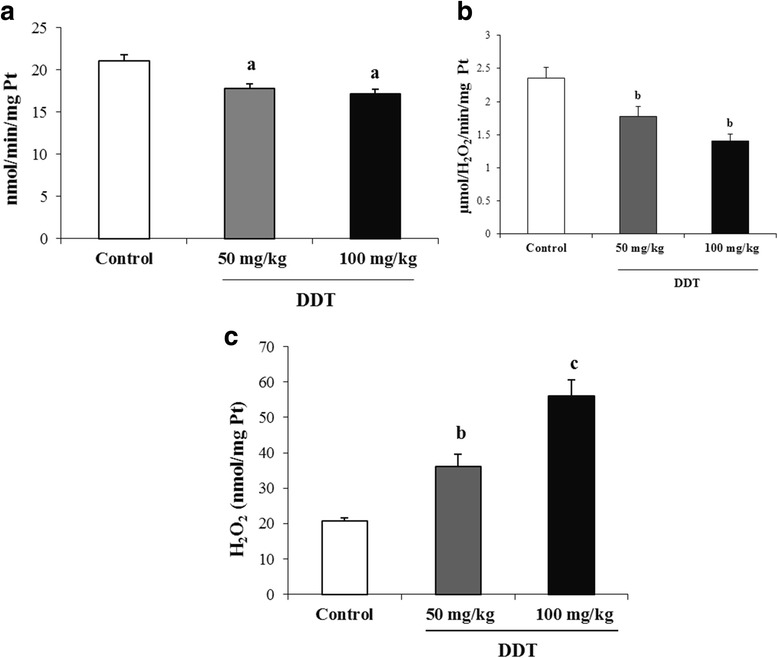
Effect of DDT on antioxidant enzyme activities in testis of adult rat. Superoxide dismutase activity (**a**), catalase activity (**b**) and hydrogen peroxide production (**c**). Each value is the mean of 8 determinations with standard error (SEM). DDT treatment was preformed as described in Methods. ^a^ : *p* < 0.05 compared with controls (Tukey’s multiple comparison post hoc test). ^b,c^ : *p* < 0.01 compared with controls (Tukey’s multiple comparison post hoc test)

The antioxidant enzymes activities in testis are presented in Fig. 3. DDT treatment significantly reduced SOD (17.74 ± 0.62 and 17.14 ± 0.51 nmol/min/mg protein (Pt) respectively with 50 and 100mg of DDT/kg versus 21 ± 0.7 nmol/min/mg Pt) and CAT (1.77 ± 0.15 and 1.40 ± 0.10 μmol/H_2_O_2_/min/mg Pt respectively with 50 and 100 mg of DDT/kg versus 2.35 ± 0.15 μmol/H_2_O_2_/min/mg Pt) activities in a dose-dependent fashion (Fig. 3a and b). However, the level of H_2_O_2_ in testis increased from 20.8 ± 0.86 to 36.2 ± 3.36 and 55.97 ± 4.72 nmol/mg Pt, respectively for 50 and 100 mg of DDT/kg (Fig. 3c).

### Glutathione-dependent enzymes and reducing power

Glutathione-dependent enzymes and reducing power in testis are presented in Table 1. The Gpx activity decreased in animals exposed to DDT by 24% and 61.5% of control, respectively for 50 and 100 mg/kg (Table 1). The GR activity was significantly decreased in treated rats compared to control group (22.5 ± 1.76 and 16.71 ± 0.78 U/mg Pt respectively with 50 and 100mg of DDT/kg versus 42.37 ± 2.02 U/mg Pt). The GST activity in testis was not affected in the 50 mg DDT/kg group but was significantly decreased in 100 mg DDT/kg group (58.7 ± 3.3 U/mg Pt versus 107.4 ± 2.8 U/mg Pt). DDT treatment induced a dose-dependent increase in GSSG levels (Table 1). This increase reached 29.7% and 78.5% of controls for 50 and 100 mg/kg, respectively. In contrast, the GSH level was significantly decreased in treated rats compared to control group (Table 1). This decrease reached 24.5% and 52.5% of controls for 50 and 100 mg of DDT/kg, respectively. The ratio between concentrations of GSSG and GSH is a valuable marker, characterizing cellular redox status. Thus, exposure to DDT significantly increased the ratio GSSG/GSH in treated rats compared to control group (Table 1). This increase reached 100% and 322.5% of controls for 50 and 100 mg/kg, respectively.

**Table 1:** Effect of DDT treatment on glutathione-dependent enzymes and reducing power in testis of adult rat

Parameters	Control	DDT 50 mg/kg	DDT 100 mg/kg
Gpx (U/mg Pt)	76.83 ± 1.99	58.26 ± 2.25^a^	29.6 ± 1^b^
GR (U/mg Pt)	42.37 ± 2.02	22.5 ± 1.76^a^	16.71 ± 0.78^b^
GST (U/mg Pt)	107.45 ± 2.82	109.86 ± 4.71	58.75 ± 3.29^a^
GSH (nmol/mg Pt)	59.36 ± 2.87	44.81 ± 1.53^a^	28.18 ± 2.63^b^
GSSG (nmol/mg Pt)	5.59 ± 0.21	7.25 ± 0.32^a^	9.98 ± 0.3^b^
GSSG/GSH (%)	0.089 ± 0.003	0.178 ± 0.004^a^	0.376 ± 0.036^b^

Each value is the mean of 8 determinations with standard error (SEM). DDT treatment was preformed as described in Methods. ^a, b^ : *p* < 0.05 compared with controls (Tukey’s multiple comparison post hoc test)

### Fragmentation of DNA induced by DDT

**Fig. 4:**
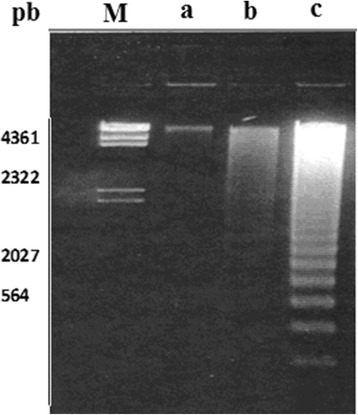
In vivo DDT apoptotic DNA laddering in testis. Testicular DNA was isolated, subjected to agarose gel electrophoresis and stained with ethidium bromide as described in the text. Each lane contained 4μg of DNA. Lanes right to left: **a** control, no treatment; **b** treated rats with 50mg of DDT/kg; **c** treated rats with 100mg of DDT/kg. Lane M: 1kb molecular weight DNA ladder marker

Evidence of DNA fragmentation in rat testis treated with DDT was obtained by ethidium-bromide agarose gel electrophoresis (Fig. 4). DNA isolated from the testicular tissues of rats after administration of DDT to animals for 10 days showed degradation into oligonucleotide fragments forming a clear laddering pattern of apoptosis when separated by 1.5% agarose gel electrophoresis (Fig. 4, lane b and c), whereas DNA fragmentation is negligible in testis for control (Fig. 4, lane a).

### TUNEL assay

**Fig. 5:**
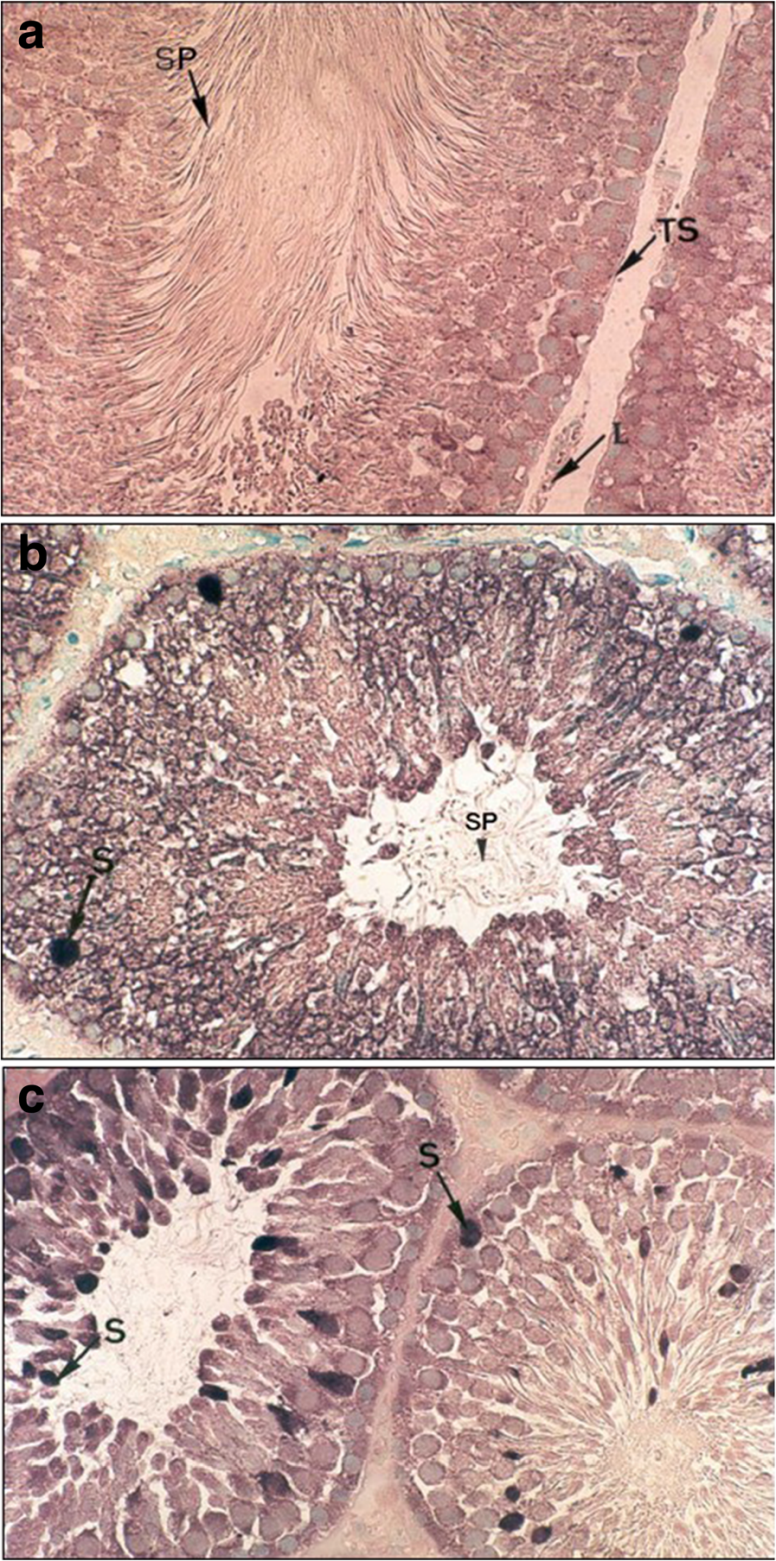
Detection of apoptotic cells in the testis of rats as revealed by TUNEL assay. Seminiferous tubule from control showed no apoptotic cells (Photo **a**). Seminiferous tubules from DDT- treated rats with 50 mg/kg (Photo B) and 100 mg/kg (Photo **c**) showed TUNEL-positive germ cells. Rats received an ip injection of 50 or 100 mg/kg body weight of DDT during 10 days. TS, Seminiferous tubule; SP, spermatozoa; S, spermatocyte cells; L, leydig cells. Magnification: ×400 (**a, b, c**)

Apoptosis was characterized by a TUNEL technique that specifically detects apoptotic cells in testes. Untreated rats showed no apoptotic cells in the seminiferous tubule (Fig. 5, Photo a), whereas positive staining in germ cells was found after treated with 50mg of DDT/kg (Fig. 5, Photo b). With 100 mg of DDT/kg, strong positive staining was observed in germ cells (Fig. 5, Photo c). The apoptotic index grew 8.2 fold (*p* < 0.01) and 23.2 fold (*p* <0.01) in treated rats with 50 and 100 mg of DDT/kg, respectively compared to control (Table 2).

**Table 2:** Effect of DDT treatment on apoptotic index in testis of adult rat

	Apoptotic index (%)
Control	0.55 ± 0.09
DDT 50 mg/kg	4.5 ± 0.92^a^
DDT 100 mg/kg	12.78 ± 1.54^b^

Each value is the mean of 8 determinations with standard error (SEM). DDT treatment was preformed as described in Methods. ^a,b^ : *p* < 0.05 compared with controls (Tukey’s multiple comparison *post hoc* test)

## Discussion

The purpose of this study was to investigate how the p,p’-DDT treatment induced oxidative stress and the mechanisms involved in DDT-induced apoptosis in testis. Lipid peroxidation is an identified cell damage mechanism in plants and animals, and it is used as an indicator of oxidative stress in cells and tissues. Our results showed that exposure of rats to 100mg of DDT/kg b.wt, during 10 consecutive days, significantly increased MDA level in the testis. Our finding was in accordance with another study carried out in rats and which have also reported a high level of lipid peroxidation in the testis of DDE- treated rats [36]. It was reported that DDT induced reactive oxygen species (ROS) generation in different animal tissues, including human cells [19, 37]. It is well documented that male reproductive organs are particularly susceptible to the deleterious effects of ROS and lipid peroxidation, which ultimately lead to impaired fertility [38]. Increased LPO during spermatogenesis leads to tissue damage [39], impaired membrane function, decreased membrane fluidity, altered structural integrity and inactivation of several membrane bound enzymes [40]. Previous studies reported the enhanced production of ROS in testis exposed to p,p’-DDE [21, 41]. MTs are members of a family of low molecular weight proteins rich in cysteine that play a key role in transport of essential heavy metals, detoxification of toxic metals and protection of cells against oxidation stress. Our result showed that the levels of MTs decreased in a dose-dependent manner in testis of DDT-treated rats. The inhibited level of MTs is closely associated with increased formation of ROS and reactive nitrogen species, respectively. Excessive production of these harmful substances along with a reduction in anti-oxidants could reduce the level of MTs in testis [42]. Antioxidant enzymes, such as SOD and CAT, are essential parts in the cellular defense against free radical–mediated tissue or cellular damage. Our results showed a decrease in the level of SOD and CAT activities while H_2_O_2_ production increased in the testis of DDT-treated rats. Similarly, recent studies showed that exposure to p,p’-DDE for 10 consecutive days decreased SOD activity in testis [36, 41]. The increase of hydrogen peroxide levels may be due to reduced SOD and CAT activities in testis. This condition could be favorable to hydroxyl radical formation which may lead to lipid peroxidation [43]. Besides, SOD is known to catalyse the dismutation of superoxide anions to H_2_O_2_ and molecular oxygen, while CAT has been shown to be responsible for the detoxification of H_2_O_2_ [44]. The reduction in CAT activity may reflect less capacity of testicular mitochondria and microsomes to eliminate H_2_O_2_ produced in response to DDT [45]. It is also known that CAT protect SOD against inactivation by H_2_O_2_ and that SOD, in turn, protects CAT against superoxide anions. The balance of these enzyme systems may be essential to testicular health. Hence, the significant reduction in enzyme activities, accompanied by marked increase lipid peroxidation, may reflect adverse effects of DDT on the antioxidant system [41]. Therefore, the decrease in SOD and CAT activities may explain the early-elevated ROS levels, since it was a crucial enzyme involved in the detoxification of ROS. Moreover, our results showed that p-p’-DDT administration decreased testicular Gpx, GR, GST and GSH activities while increased the ratio GSSG/GSH, which led to the production of free radicals and causing LPO [46]. GSH is one of the most important non-enzymatic antioxidant against cellular damage produced by ROS [47]. GSH supplementation has been shown to have a protective action against seminal plasma lipid peroxidation, and it has been implicated in the treatment of male infertility [48]. The decreased glutathione concentration may be explained by the adverse effect of ROS which can decrease synthesis and/or reduce transport into the testis (because it is well known that most GSH is synthesized by liver), or accelerate degradation or enhance export of oxidized form [49]. It cannot be excluded that the system(s) of synthesis and transport of GSH could be affected by exposure to DDT. GR is involved in the supplementation of GSH to spermatogenic cells [50]. GSH is also GST co-substrat. GST catalyzes the conjugation of reduced glutathione with a variety of endogenous compounds and xenobiotics [51]. Therefore a depression in GSH levels together with GST activity makes the cells more susceptible to the attack by toxic compounds [52]. In the present study, the decrease in GPx, GST and GSH activities, accompanied by the increase of GSSG/GSH ratio and MDA levels, supports that oxidative stress is produced due to DDT administration. Apoptosis is a genetically regulated cellular suicide mechanism in which multiple signaling pathways are implicated [53]. Among them, oxidative stress is an important event which may affect different macromolecules and components of the cells, triggering the activation of several antioxidant response genes and mechanisms [53]. The oxidative stress could be associated with severe damage to DNA. Previous study revealed that exposure to DDT induced DNA single-strand breaks [54]. Recently, it was reported that exposure to p,p’-DDE induced DNA damage in Sertoli cells, which might account for subsequent development of apoptosis [21, 36, 41]. In this study, the DNA isolated from testicular tissues of DDT-treated rats showed degradation into oligonucleotide fragments forming a clear ladder pattern. In addition, histological examination of testicular tissue by the TUNEL method showed that apoptosis cells occurred in the germ cells of DDT-treated rats. Also, the apoptotic index was significantly increased in testis of DDT-treated rats. Apoptosis is a complex event regulated by a well-tuned balance of inducer and repressor factors, such as the Bcl-2 family, which is a pivotal integrator of survival and death signal. In addition, the Fas system is a widely recognized apoptosis signal transduction pathway in which a ligand-receptor interaction triggers the cell death pathway [55]. Fas is a surface receptor that triggers apoptotic cell death when cross-linked by FasL [56]. Ligation of FasL to Fas in the cell membrane triggers activation of caspase-8. Once activated, caspase-8 transduces a signal to effector caspases, including caspases 3, 6, and 7, and eventually leads to the hydrolysis of cytosolic and nuclear substrates [57]. Previous studies showed that p,p’-DDE could induce apoptosis of Sertoli cells through a FasL-dependent pathway including nuclear translocation of NF-*κ*B, increase of the FasL expression, and activation of the caspase 8 and 3 [21, 36]. Recently, it was reported that p,p’-DDT activated NF-*κ*B/FasL pathway and mitochondrial pathway in human liver cells which were mediated by ROS. Moreover, it has been demonstrated that the excess or deprivation of hormones such as FSH and testosterone can lead to cellular apoptosis in the testis [58]. It has been shown that both extrinsic and intrinsic apoptotic death pathways are operative in the germ cells following decrease in FSH and testosterone levels; therefore, FSH and testosterone maintain spermatogenic homeostasis by inhibiting death signals for the germ cells [59]. In our earlier study, serum FSH and LH levels were significantly increased and testosterone levels were decreased in rats exposed to 50 and 100mg of DDT/kg for 10 days [17]. It is possible that the decreased testosterone levels and the increased FSH levels in response to DDT exposure stimulates caspase activity and produces DNA fragmentation in germ cells [60]. Fewer study elucidated the mechanism of DDT-induced apoptosis in testis. So, in this study, we have shown for the first time that p,p’-DDT treatment induced apoptosis in germ cells. These findings suggested that p,p’-DDT-induced apoptosis of germ cells through mitochondria-mediated and FasL-dependent pathway. It is possible that different stimuli such as DNA damage or increased ROS level caused by DDT might trigger Bax activation via acting diverse molecules such as p53, and Fas system. Activation of Bax protein leads to the formation of pores in the mitochondria and results in the collapse of the electro chemical gradient across the mitochondrial membrane, then cytochrome *c* is released into cytoplasm where it is associated with procaspase-9/Apaf-1. This complex, in turn, activates a downstream caspase program that ultimately leads to apoptotic cell death [61].

## Conclusions

In conclusion, the results obtained from the present study demonstrate that the sub-acute treatment of p,p’-DDT causes DNA fragmentation and apoptotic cell death in testis probably mediated by oxidative stress which leads to the adverse toxic effects of DDT on male reproduction of rats. Further studies are needed to elucidate the expression and/or activity of pro and anti-apoptotic proteins in testicular cells after exposure to DDT.

## Abbreviations

CAT: Catalase
GR: Glutathione reductase
GSH: Reduced glutathione
GSSG: Oxidized glutathione
GST: Glutathione S-transferase
H_2_O_2_: Hydrogen peroxide
LPO: Lipid peroxidation
MDA: Malondialdehyde
MTs: Metallothioneins
PBS: Phosphate-buffered saline
ROS: Reactive oxygen species
SOD: Superoxide dismutase
